# Exploring relationships between HIV programme outcomes and the societal enabling environment: A structural equation modeling statistical analysis in 138 low- and middle-income countries

**DOI:** 10.1371/journal.pgph.0001864

**Published:** 2023-05-09

**Authors:** Dejan Loncar, Jose Antonio Izazola-Licea, Jaya Krishnakumar

**Affiliations:** 1 Institute of Global Health, University of Geneva, Geneva, Switzerland; 2 ThinkWell, Geneva, Switzerland; 3 UNAIDS, Geneva, Switzerland; 4 Institute of Economics and Econometrics Geneva School of Economics and Management, University of Geneva, Geneva, Switzerland; University of Minnesota Medical School Twin Cities, UNITED STATES

## Abstract

Countries worldwide have attempted to reduce the incidence of HIV and AIDS associated deaths with varying success, despite significant progress in antiretroviral treatment (ART) and condom use. A chief obstacles is that key populations affected face high levels of stigma, discrimination and exclusion, limiting the successful response to HIV. However, a gap exists in studies demonstrating the moderation effects of societal enablers on overall programme effectiveness and HIV outcomes using quantitative methods.Structural Equation Modeling was used for 138 countries covering a 12-year period to examine how the unfavorable societal enabling environment, including stigma and discrimination, unfavorable legal environment and lack of access to societal justice, gender inequality and other unfavorable development situations affect the effectiveness of HIV programmes and HIV outcomes, while controlling for potentially confounding variables. The results only showed statistical significance when all four societal enablers were modeled as a composite. The findings show the direct and indirect standardized effects of unfavorable societal enabling environments to AIDS-related mortality among PLHIV are statistically significant and positive (0.26 and 0.08, respectively). We hypothesize that this may be because an unfavorable societal enabling environment can negatively affect adherence to ART, quality of healthcare and health seeking behavior. Higher ranked societal environments increase the effect of ART coverage on AIDS related mortality by about 50% in absolute value, that is -0.61 as against -0.39 for lower ranked societal environments. However, mixed results were obtained on the impact of societal enablers on changes in HIV incidence through condom use. Results indicate that countries with better societal enabling environments had fewer estimated new HIV infections and fewer AIDS-related deaths. The failure to include societal enabling environments in HIV response undermines efforts to achieve the 2025 HIV targets, and the related 2030 Sustainable Development indicator to end AIDS, even if sufficient resources are mobilized.

## Introduction

The global response to HIV has been defined by a series of specific programmes that provide testing, treatment, prevention, health sector strengthening and additional support activities, such as monitoring, evaluation, programme management, social mobilization, community engagement, peer education, legal and policy reform, human rights advocacy, etc. UNAIDS, in its 2011 investment framework [1], defined a series of programmes that could reduce AIDS-related mortality and HIV-incidence, as well as the critical enablers classified as programme and societal enablers.

There are clear 2030 goals to reduce new HIV cases, AIDS related deaths and stigma and discrimination close to zero, or 90% reductions compared to 2010 values, for which there needs to be a supporting enabling environment (societal enablers) and functioning health and social systems (System and Service enablers). The UNAIDS Strategy (2021–2026) has incorporated a new set of targets for 2025, including those addressing financial needs and an updated framework showing basic programmes and societal enablers. These targets are intended to guide and influence countries, donors, and implementing organizations. The study presented in this paper is part of a series of papers in the Public Library of Science (PLoS) journals and supported the 2025 HIV target-setting process [2]. The 2021 UNAIDS update of the societal enablers framework expanded and further defined “societal enablers’’ to indicate the need for engagement of the society more broadly in order to exert structural change [3].

**Fig 1:**
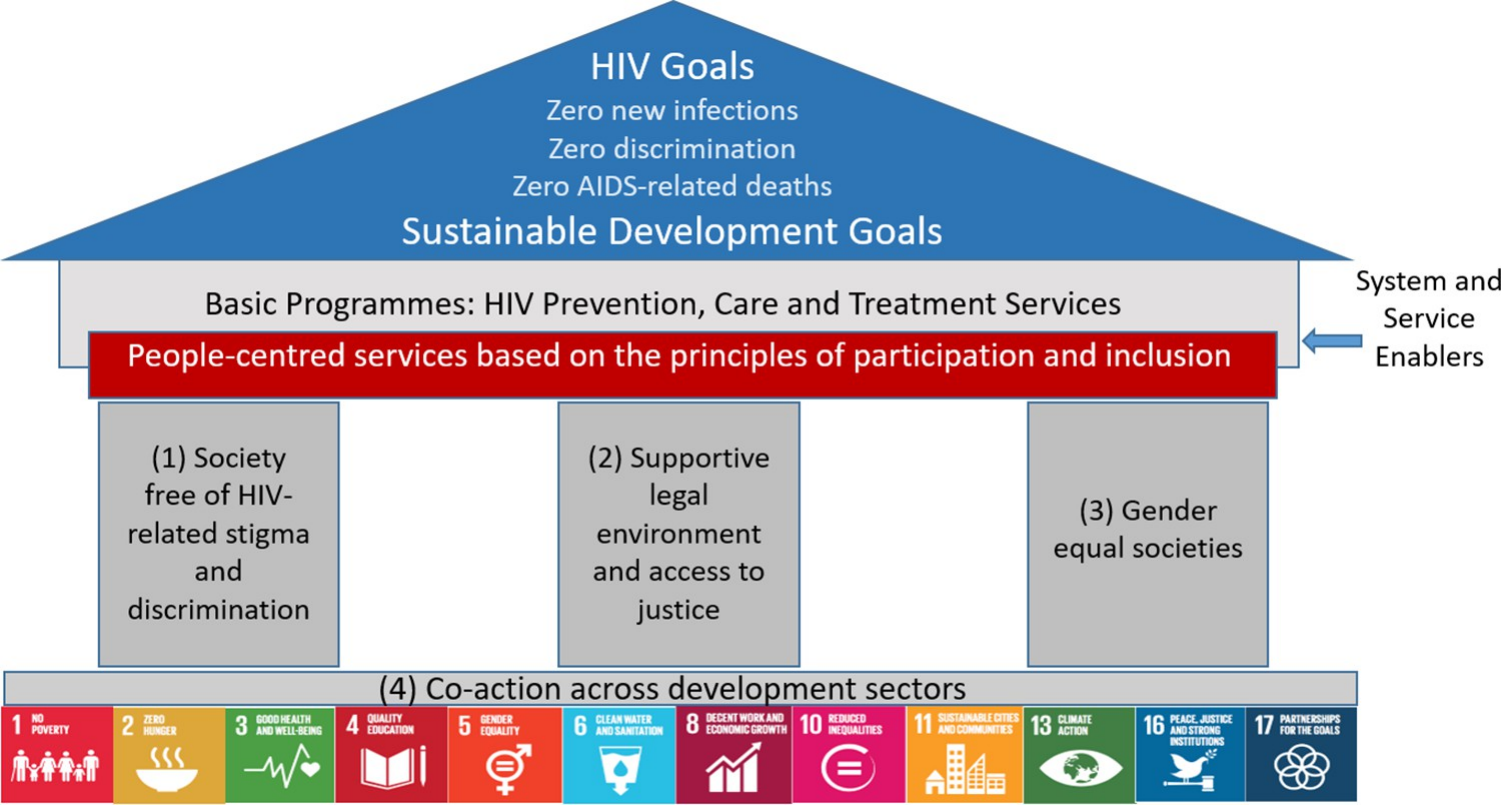
Societal enablers framework.

Societal enabling interventions in the HIV arena can be defined as those that address societal structural issues that hamper HIV responses. They fall under four main categories [3]: (1) Society free of HIV-related stigma and discrimination, (2) Supportive legal environment and access to justice, (3) Gender equal societies and (4) Coaction across development sectors (Fig 1). Not all societal enablers that are relevant to the effectiveness of HIV programmes fall exclusively under the direct area of influence of HIV or health programmes but may be part of broader development areas.

This study uses a Structural Equation Model (SEM) to estimate the effects of societal enablers on HIV programme effectiveness. The Methods section below explains the suitability of the SEM framework to answer this research question as well as its different components.

The hypothesis that guided the research came from a systematic review of published literature and the UNAIDS framework, including the theoretical concepts. The premise is that an unfavorable societal enabling environment (unfavorable S.E.E.) negatively modifies the effectiveness of key HIV services, such as antiretroviral treatment (ART) or condom use. The study focuses on these two HIV services given that there is not enough reliable cross-country information for other components of HIV programmes such as Voluntary medical male circumcision (VMMC), Pre-Exposure Prophylaxis (PrEP), partner reduction and the other metrics of treatment including achievement of viral suppression. That is, there was not enough data on viral suppression to assess the concept of treatment as prevention relating to individuals with undetectable viral load who would not transmit HIV, i.e. undetectable equals untransmissible or U = U.

Effects on two HIV outcomes were observed—AIDS related mortality and HIV incidence change over 10 years. HIV Incidence change was used instead of HIV incidence due to small annual change of HIV incidence. The authors postulate that there is a significant difference in the effectiveness of HIV programmes on HIV-related outcomes between countries with lower and higher scores on societal enabling environments. It is suggested that, when ART coverage is held constant, countries with a more favorable societal enabling environment will be able to achieve lower levels of AIDS-related mortality than countries with an unfavorable societal enabling environment. Similarly, at same levels of condom use, more favorable societal enabling environments contribute to lower levels of HIV incidence.

## Materials and methods

### Study design and model specification

Given the existing definition of societal enabling environment [3], it is clear that this theoretical concept cannot be directly observed by any single measure. However, the authors speculate that its different aspects can be captured through other multiple indicators. Due to the inherently negative nature of the indicators which we have used in our model (e.g. discrimination of people living with HIV is a negative occurrence), the sub-constructs were also inherently negative (e.g. several indicators which related to discrimination and stigma of key populations, ended up constructing a “Stigma and discrimination” sub-construct, all of which are negative events). Thus, the unfavorable societal enabling environment is posited to be a latent composite construct made up of four sub-constructs: stigma and discrimination, unfavorable legal environment and lack of access to societal justice, gender inequality, and other unfavorable development situations. These correspond to the societal enablers in Fig 1. Each of these four societal enabler sub-constructs is itself a latent variable and needs to be measured through several observable variables called societal enabler indicators (S1 and S2 Tables). For example, the variable “other unfavorable development situation” consists of variables such as unemployment, poverty, and illiteracy [3]. Furthermore, each of these sub-constructs and their indicators feature in the Sustainable Development Goals [4].

**Fig 2:**
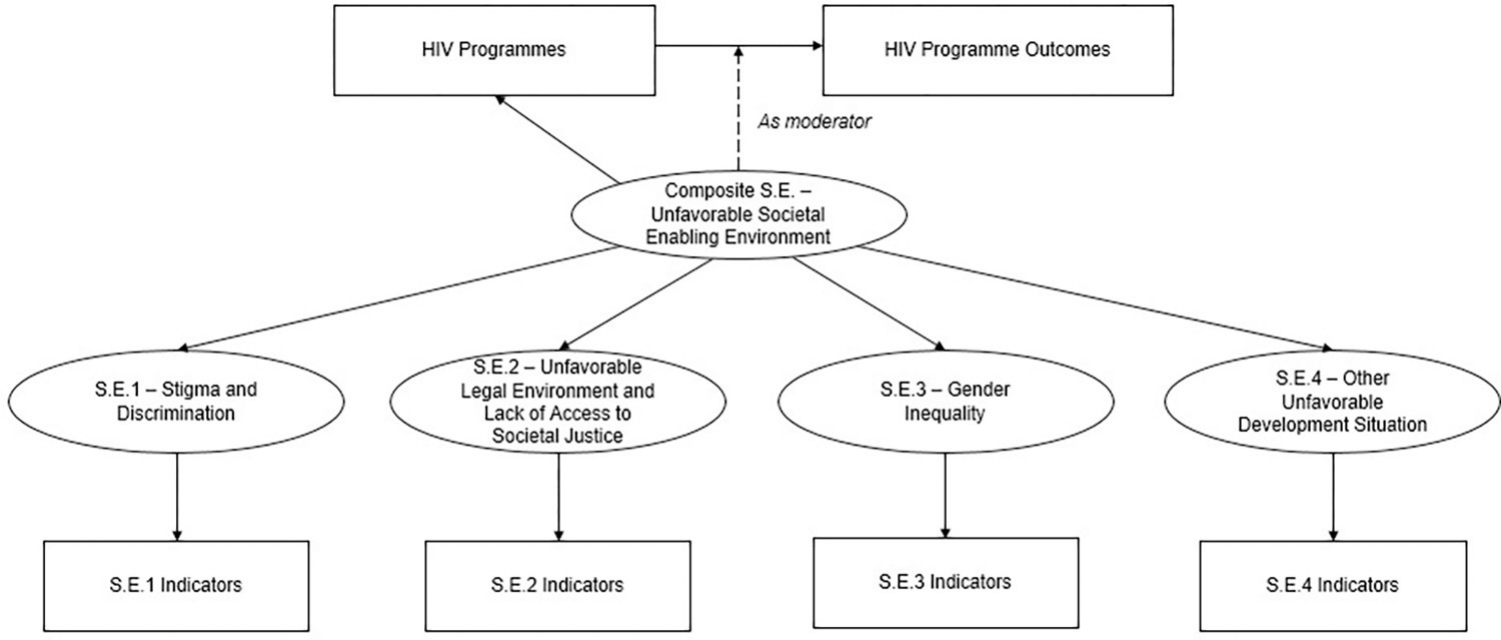
Conceptual structural model.

The model and its various components can be seen in Fig 2. In the lower part, the four ellipses represent the four latent sub-constructs and the corresponding boxes underneath, the societal enabler indicators. The aggregation of these four sub-constructs to obtain an unfavorable societal enabling environment is represented by the single ellipse named Composite Societal Enabler (Composite S.E.). At the highest level is the relationship between HIV basic programmes and HIV outcomes. The hypothesis is that the unfavorable societal enabling environment plays a key role in moderating this relationship and this is represented by the dashed arrow from the Composite S.E. to the link between HIV programmes and HIV outcomes. In addition, the Composite S.E. can have a direct effect on HIV programmes, represented by the arrow from the Composite S.E. to HIV programmes.

Therefore, this model investigates two key relationships: the effect of unfavorable S.E.E. on HIV programmes and its moderating effect on the impact of HIV programmes on HIV programme outcomes. The inclusion of the unfavorable S.E.E. at these two levels is the novel contribution of this methodology. It provides a means to verify the theoretical hypothesis that the same HIV programmes would be more effective in a favorable societal enabling environment than in an unfavorable societal enabling environment.

The analysis consisted of two phases of modeling that are sequential to each other. Firstly, based on second order confirmatory factor analysis (CFA), estimations were determined for the four societal enablers and the resulting composite societal enabler. These estimations were arrived at using theory and evidence-based indicators from the updated UNAIDS framework [3], relevant publications and Global AIDS Monitoring data. The Second Order CFA [5] is a statistical method used to confirm that the theorized construct in a study loads into a certain number of underlying sub-constructs. A Second Order CFA can be particularly useful in situations where a theoretical construct is thought to consist of multiple, interrelated sub-constructs. By using this statistical method, it can be tested whether these sub-constructs are empirically supported and whether they are related to the overall construct of interest. The results of a Second Order CFA can help to provide more insight into the structure and composition of the construct being studied, which can ultimately help to guide future research and theory development. Secondly, this study provides an assessment of the direct, indirect, and moderation effects of the estimated composite societal enabler on HIV outcomes, by using Structural Equation Modeling (SEM). SEM is warranted whenever the concepts under study are not directly observable and need to be postulated as latent variables, measured by multiple indicators, before being entered in the key structural relationship under study—as is the case for the unfavorable societal enabling environment. Similar analysis has been conducted previously [6] whereby SEM was used to estimate the indirect associations of enablers to treatment coverage and the subsequent associated impact on AIDS related mortality.

This study mimics the UNAIDS-accepted Goals Model [7], applying SEM techniques, and adding the societal enabler component. The Goals Model application, which is a part of the Spectrum software package, uses data and information from surveys and other surveillance information and calibration models to generate projections of incidence, prevalence, and resource needs [8]. In the Goals model, AIDS-related mortality rates for people on ART are based on analysis of treated populations conducted by the IeDEA Consortium. Mortality rates are differentiated by sex, age, CD4 count at ART initiation, duration of treatment and region. CD4 count at initiation is already included in the SEM and the distribution of people on ART by age and sex is similar across countries and unlikely to be significantly affected by societal enablers. The regional variable was not included in the SEM model, thus variability originally captured in GOALS through this variable, might be partly captured through an unfavorable societal enabling environment.

Incidence in the Goals model is determined by a large number of factors, including condom use. The effectiveness of condom use in preventing HIV transmission per act is fixed in the model based on studies, at 80%. However, the overall impact of condom use depends on levels of use by population group (sex workers and clients, men who have sex with men, people who inject drugs, people with multiple partners, stable couples) so any relationship to societal enablers would work even better by affecting condom use rates in each group or the relative contribution of each group to overall incidence. Such data disaggregation is not available at the moment, hence this approach could not be implemented.

### Data collection and management

To estimate the four dimensions of societal enablers, an initial set of 98 indicator variables was identified based on thorough research of the UNAIDS framework, relevant publications [3, 6] and various data sources (S1 and S2 Tables). The chief constraint of this study was the unavailability and scarcity of data. In particular, it was challenging to find indicators from a large number of countries with comparable and reliable data. Out of the 98 indicators, 10 were unavailable, and hence could not be used in the analysis, leaving 88 indicators for potential use.

Regarding the time periods, data were missing for several years from the interval 2010–2019 for several indicators. Hence, focus has been placed on two years—2012 and 2017, as a median of the two 5-year periods, 2010–2014 and 2015–2019. Of the 88 indicator variables available for the years 2012 and 2017 combined, the average percentage of missing data points, before imputation, was 79.9%. This percentage is too high to perform any reliable form of multiple imputation or to use the full information maximum likelihood (FIML) option to handle missing data. In order to increase available data points in years 2012 and 2017, data from adjacent years was used, the rationale being that the societal environment only changes slowly over time. Imputation methods were used in two stages, based on specifics of each variable:

1. Relevant data points (from the same country and indicator) in years ranging from 2010 to 2014 were used to impute the missing data for the year 2012. If the relevant data was available for only one year from 2010, 2011, 2013 and 2014, the value for that year was used to impute the missing value for 2012. If the relevant data was available for two or more years from 2010, 2011, 2013 and 2014, then the average of available values was used to impute the missing value in 2012. The same process was used for imputing missing data points for 2017, using available relevant data from adjacent years 2015, 2016, 2018, 2019. Using this method, 29.5% of missing data was imputed for years 2012 and 2017 combined, leaving a total of 50.4% missing data points.
2. For indicators measured on a continuous scale, such as literacy rate or gender parity index for example, the second stage of imputation methods was applied. If the country has a missing value for the years 2012 or 2017 after the first stage of the imputation process, the average regional change rate for the variable of interest between years 2012 and 2017 was used to impute the missing value. Using the second method of imputation, 5.2% of data was imputed, leaving us with a total of 45.2% of missing data.

Similar imputation methodology was used in earlier publications [9, 10], where the missing financial, socio-economic and risk-factor data was imputed with the sub-regional or regional average of the given variable of interest.

After imputation, indicators that were very similar theoretically (for example, same question with male/female/total respondents) were identified. For each of the four societal enablers the most relevant variables (i.e. totals) with the most complete data were kept, while others were removed. Subsequently, a stepwise exclusion method was used to fit the model and exclude indicators with very low factor loadings that are considered to have low association with corresponding societal enablers.

In the case of some societal enablers, in particular S.E.1 Stigma and discrimination and S.E.3 Gender inequality, there was very limited indicator data available. For this reason, and in line with the UNAIDS framework and available evidence, some indicators deemed as essential for constructing these societal enablers were kept, despite the corresponding low factor loadings or a high percentage of missing values. This was the case with Discriminatory attitudes towards PLHIV, Internalized stigma among PLHIV and Women 15–49 who experienced violence from their partner.

Finally, 19 indicators of societal enablers made it into the set used in the second order CFA model. The S1 Table presents the missing data percentage after each imputation stage for years 2012 and 2017 combined, sources of data, and other specific information in detail for the 19 S.E. indicators, 3 HIV programme variables, and 2 HIV outcomes used in our SEM model. The S2 Table contains information about remaining S.E. indicators that were considered, but not included in the model. Due to the large number of missing observations (different years available for different countries/indicators), it was only possible to estimate societal enablers and the composite S.E. for 2 years (2012 and 2017), as explained above.

In order to create a structure with HIV Programmes and HIV Outcomes (HIV incidence change and AIDS-related mortality) data from 2010 to 2021, a linear trend was used to estimate values in the missing years of the composite S.E. The rationale for this decision was that the societal enabling environment of a specific country does not change as quickly as other variables, such as HIV programmes [6]. Thus, 1656 observed data points were identified (from 138 countries over 12 years).

The HIV programme outcome variables used were the estimated annual HIV-incidence percent change and AIDS-related mortality obtained from the GOALS model due to the lack of valid and reliable observable and reported data from countries and due to the soundness of the GOALS country-by-country estimates.

When it comes to HIV incidence change, in certain countries with low baselines the high increase of HIV Incidence in recent years created outliers. For example, Pakistan and Tajikistan had an 80-fold HIV incidence increase from 2000 to 2010. In the year 2000, the countries had a markedly low number of cases, which, when looking at 2010 numbers seemed to have a large percentage increase, even though the numbers for 2010 were still low. This relates to thirty-two out of 1656 data points (under 2% of outliers), which were treated as missing values. In the subsection Statistical Analysis there is an explanation of model fit statistics, missing data handling methods and other details about the model applied.

### Statistical analysis

The following statistics were used to evaluate model fit: (1) comparative fit index (CFI), and (2) root mean square error of approximation (RMSEA). While these models widely apply thresholds of greater than 0.95 (CFI) and less than 0.08 (RMSEA) to indicate a good fit, for this model values of greater than 0.9 and less than 0.1 were applied in light of the limitations of the data and based on earlier precedent [11].

As a measure of reliability, composite reliability (CR) was evaluated. Values of CR over 0.7 were treated as indicative of good reliability [12]. As a measure of convergent validity, average variance extracted (AVE) was evaluated. Values of AVE over 0.5 were taken as indicative of good validity [12]. All analyses were conducted using SPSS AMOS version 21 software.

In order to handle missing data, the second order CFA and SEM models were estimated using the Full information maximum likelihood approach (FIML). For the second order CFA model, FIML did not provide model fit statistics, hence the Stochastic regression imputed dataset was used to obtain model fit statistics. Sensitivity analysis showed that the FIML and Stochastic regression imputation methods of handling missing data give very similar results.

In the two-stage approach [13, 14], the latent construct scores—Composite S.E. and four S.E. were first calculated and saved (from the CFA depicted by the bottom half of Fig 2). Subsequently, the interaction term was built as the element-wise product of standardized observed values of HIV programme variables and standardized estimated score of Composite S.E. Then, this interaction term and the latent score of the composite societal enabler and HIV programmes were used as explanatory variables in a structural equation model with HIV Outcomes as dependent variables (the top half of Fig 2). The magnitude of direct and indirect effects of societal enablers on HIV Incidence change and AIDS-related mortality was estimated from the SEM. Total effects were calculated as the sum of direct and indirect effects.

Using standardized regression coefficients obtained from the SEM model, the HIV outcomes were predicted for two fixed levels of the composite societal enabler, the 25th and 75th percentiles, representing the midpoints of the less and more favorable societal enabling environments respectively. The results were graphically presented to visualize the moderation effect of the composite societal enabler in the relationship between HIV programmes and outcomes.

As a final step, multi-group moderation analysis was applied to assess the differential impact of HIV programmes on HIV outcomes due to the differences in S.E.E. The countries were divided into two groups by the median value of the “Unfavorable Societal Enabling Environment”. The first group included the 50% of countries with more favorable environments, and the second 50% of countries with less favorable environments.

## Results

The analysis uses an unbalanced dataset that contains relevant data from 138 low- and middle-income countries over a 12-year period (2010–2021). The data is deemed as unbalanced due to the missing observations for many periods for several countries. The dataset includes 47 countries in sub-Saharan Africa, 26 countries in Latin America & Caribbean, 24 countries in East Asia & the Pacific, 20 countries in Europe & Central Asia, 13 countries in the Middle East & North Africa and 8 countries in South Asia. A total of 29 countries were in the low-income group, 50 countries in the lower-middle-income group and 59 countries were in the upper-middle-income group. Of the approximately 30 million people with HIV infection in 2010, 91% resided in low- and middle-income countries in 2009 [15].

### Confirmatory factor analysis

**Fig 3:**
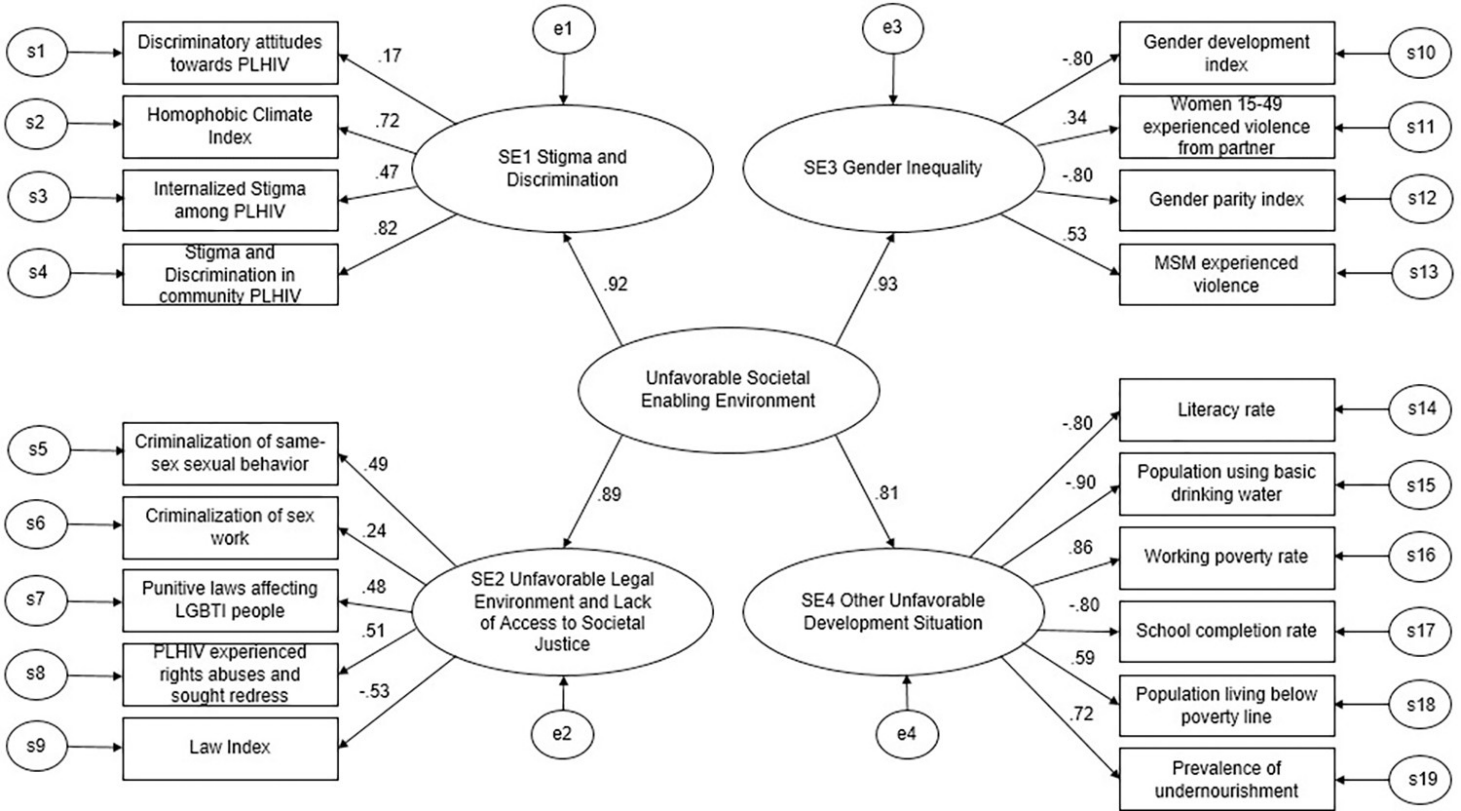
CFA model for latent variables with standardized estimates. Diagram Elements: rectangles represent observed variables (societal enabler indicators); ovals represent latent (unobserved) constructs; straight arrows point from latent constructs to observed variables and from composite societal enabler to other societal enabler variables; curved arrows are unexplained covariance among variables; small circles are residual variances of the observed variables; values are standardized regression coefficients.

High factor loadings at the second level CFA (Fig 3) showed that the Composite S.E.—unfavorable societal enabling environments had an acceptable fit with the four estimated dimensions of societal enablers. Therefore, the Composite S.E. could be used to capture the combined effect of all four societal enablers in the model applied. Factor loadings of societal enablers S.E.1-S.E.4 all have a positive sign (0.92, 0.89, 0.93 and 0.81 respectively). A higher value of each of the four societal enablers means a poorer situation of their domains and therefore a more disadvantageous situation of unfavorable S.E.E. The assumption is that an unfavorable societal enabling environment (unfavorable S.E.E.) and the 4 societal enablers negatively modify the effectiveness of key HIV services, namely antiretroviral treatment (ART) or condom use. The statistics indicated an acceptable model fit with CFI = 0.91 and RMSEA = 0.08 within the pre-established thresholds. Standardized parameters for the measurement model appear in Fig 3. All factor loadings in the model were significant (*p*-value less than 0.05), with the exception of the association between S.E.1 “Stigma and Discrimination” and the indicator “Discriminatory attitudes towards PLHIV”.

The validity and reliability of the Composite S.E. “Unfavorable Societal Enabling Environment” is indicated by its CR of 0.77 and AVE of 0.79. When it comes to the first level factor analysis, namely the four S.E. dimensions and S.E. indicators, discussing validity and reliability measures becomes more challenging. In part this is due to the fact that the four dimensions are based on theoretical coherence of concepts rather than empirical validation (e.g. an exploratory factor analysis that used all the available indicators). Thus, high correlations were found among indicators of different dimensions and this may contribute to the lower validity/reliability values at the first level. Given the limited availability of data, this approach, which relies upon a theoretical definition of societal enablers combined within the second order factor analysis, has been maintained.

### Structural equation model

**Fig 4:**
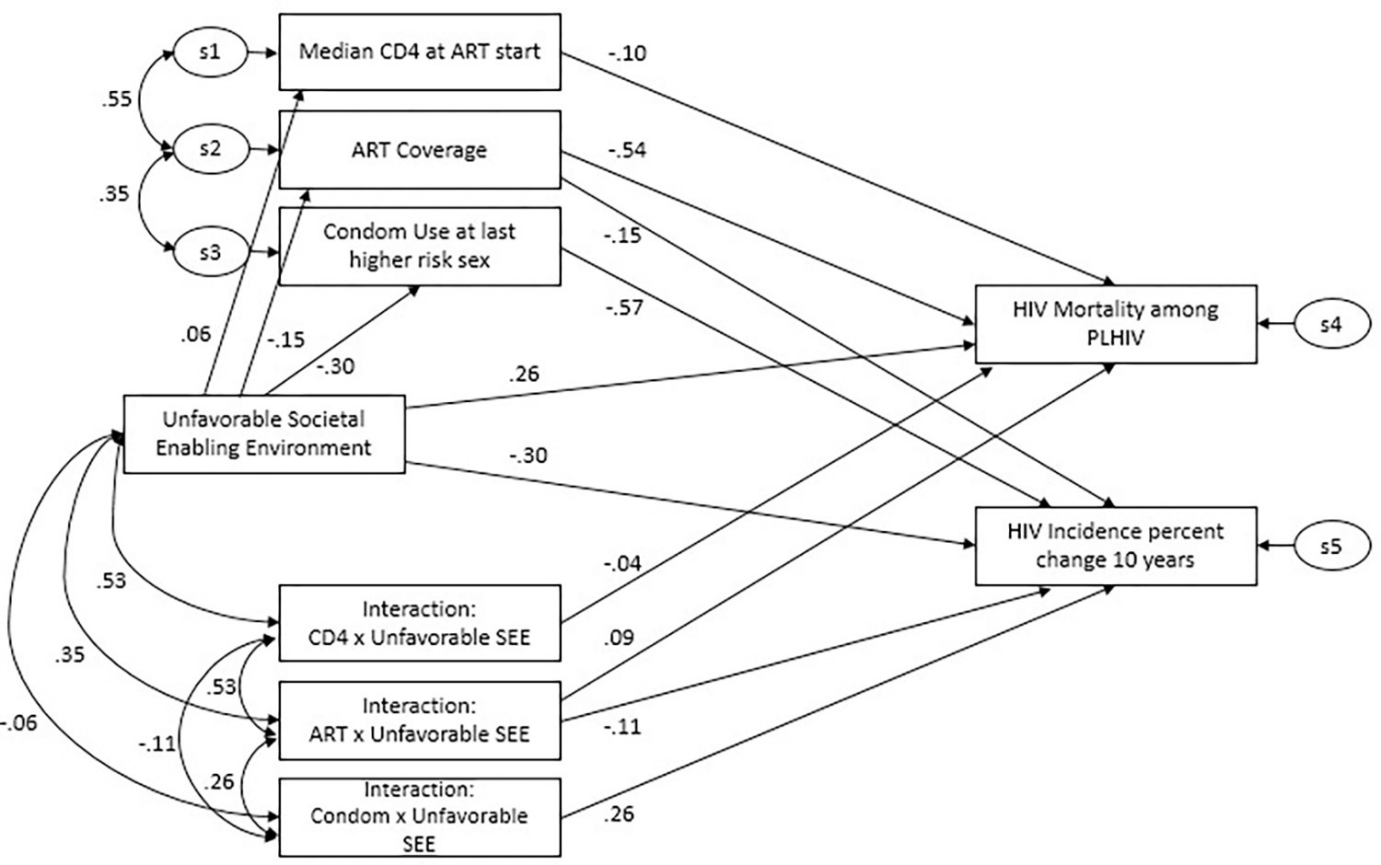
Structural equation model specifying key relationships between composite societal enabler, HIV programmes, and HIV outcomes with standardized coefficients.

The theoretical structural equation model (Fig 4) was designed to estimate the relationship between HIV programmes on outcomes, as well as the moderation effects through the unfavorable societal enabling environment. The focus of the analysis and results was on the relationships between the change in the incidence of HIV and condom use on one hand, and AIDS-related mortality among PLHIV and ART coverage on the other. The results were obtained after controlling for other important variables, such as CD4 level. The model shows a marginally acceptable fit: CFI is 0.93 and RMSEA is 0.10. All coefficients in the model were statistically significant (*p*-value less than 0.05).

Each individual dimension of the societal enabler does not have a significant effect on HIV outcomes, but the Composite S.E. has a significant effect. This can possibly be explained by the fact that each dimension only captures a partial aspect that may not be sufficient to represent what societal enabling contains in its entirety. A combination of all different aspects is required to make a social environment more or less enabling. For instance, although some aspects of a given S.E. dimension may contribute to the societally enabling environment, their positive impact may be countered by other unfavorable aspects which could be either missing in the indicators or potentially highly correlated with it, making the estimated impact of the dimension insignificant. When all the dimensions are combined, the effect of the overall environment is measured, and it was found that it needs to be enabling in order to have any significant association with HIV outcomes. As an example, a person living with HIV may have de-facto access to ART, in part due to improved legislation to prevent discrimination and access to social justice, yet the existence of stigma and/or gender inequality in accessing health services can continue to act as a deterrent and impact long-term HIV outcomes for this individual. In this case a favorable legal environment and access to societal justice may not be enough to produce an effect on HIV outcomes if gender inequality or stigma and discrimination persist and counter the positive effect of this societal enabler.

The coefficients of each relationship are indicated by the numbers on the given arrow. The direct effect of Unfavorable S.E.E. is represented by a direct arrow from Unfavorable S.E.E. to HIV incidence change and mortality (Fig 4) and measures all effects of Unfavorable S.E.E. that are not covered by HIV programme services (i.e., more incarceration, strict punitive laws, poor education, unemployment, etc.). Statistically speaking, this represents residual effects in this model. The indirect effect captures all effects of Unfavorable S.E.E. on HIV incidence and mortality affected through HIV programme services in the present analysis. For example, it captures the effect of low adherence to ART on AIDS-related mortality due to stigma and discrimination The coefficient of the direct effect of an unfavorable societal enabling environment on AIDS-related mortality is 0.26, as shown on top of the path connecting the two. The indirect effect of the unfavorable societal enabling environment to AIDS-related mortality was calculated as a sum of two effects. First, the indirect effect going through ART coverage was calculated as the product of coefficients from unfavorable S.E.E. to ART coverage (-0.15) and from the ART coverage to AIDS-related mortality (-0.54). Similarly, the indirect effect going through Median CD4 was calculated as the product of coefficients from unfavorable S.E.E. to median CD4 (0.06) and from the median CD4 to AIDS-related mortality (-0.10). Thus, the indirect effect of the unfavorable societal enabling environment to AIDS-related mortality represents the sum of the two previously calculated indirect effects, i.e. (−0.15)∙(−0.54)+0.06∙(−0.10) = 0.075.

**Fig 5:**
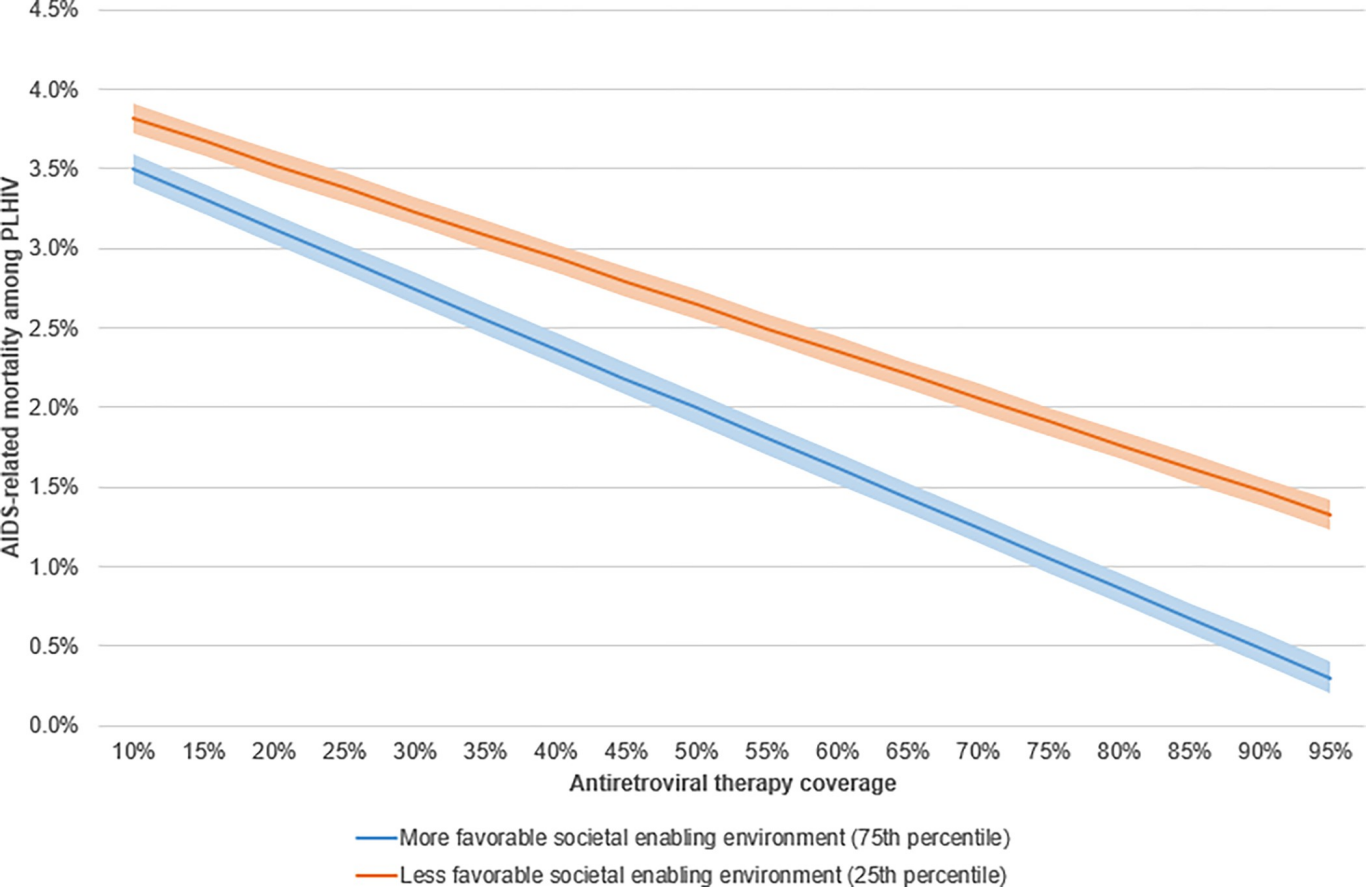
Moderating effect of unfavorable societal enabling environment on the relationship between AIDS-related mortality among PLHIV and antiretroviral coverage (2010–2021).

Next, the above findings were illustrated by examining two scenarios. Firstly, the relationship between ART coverage and AIDS-related mortality was observed, and the moderation of that relationship by the composite societal enabler (Fig 5). When the societal enabling environment is fixed at the 75th percentile, the slope is steeper compared to when it is fixed at the 25th percentile. The effects of ART coverage on AIDS-related mortality among PLHIV is greater in a more favorable societal enabling environment. In other words, the same increase in ART coverage results in greater decrease of AIDS-related mortality in a more favorable societal enabling environment. We hypothesize that this relationship may be because a more favorable societal enabling environment can positively affect adherence to ART, quality of healthcare and also seeking healthcare from the patient side. The UNAIDS goal to achieve 90% ART coverage (i.e. 95% PLHIV know their serologic status multiplied by the 95% initiated on ART equals to 90% coverage of PLHIV on ART), would have greater impact on AIDS-related mortality in more favorable societal enabling environments; in which the AIDS-related mortality rate among PLHIV was only 0.49% compared to 1.47% in less favorable environments (Fig 5).

**Fig 6:**
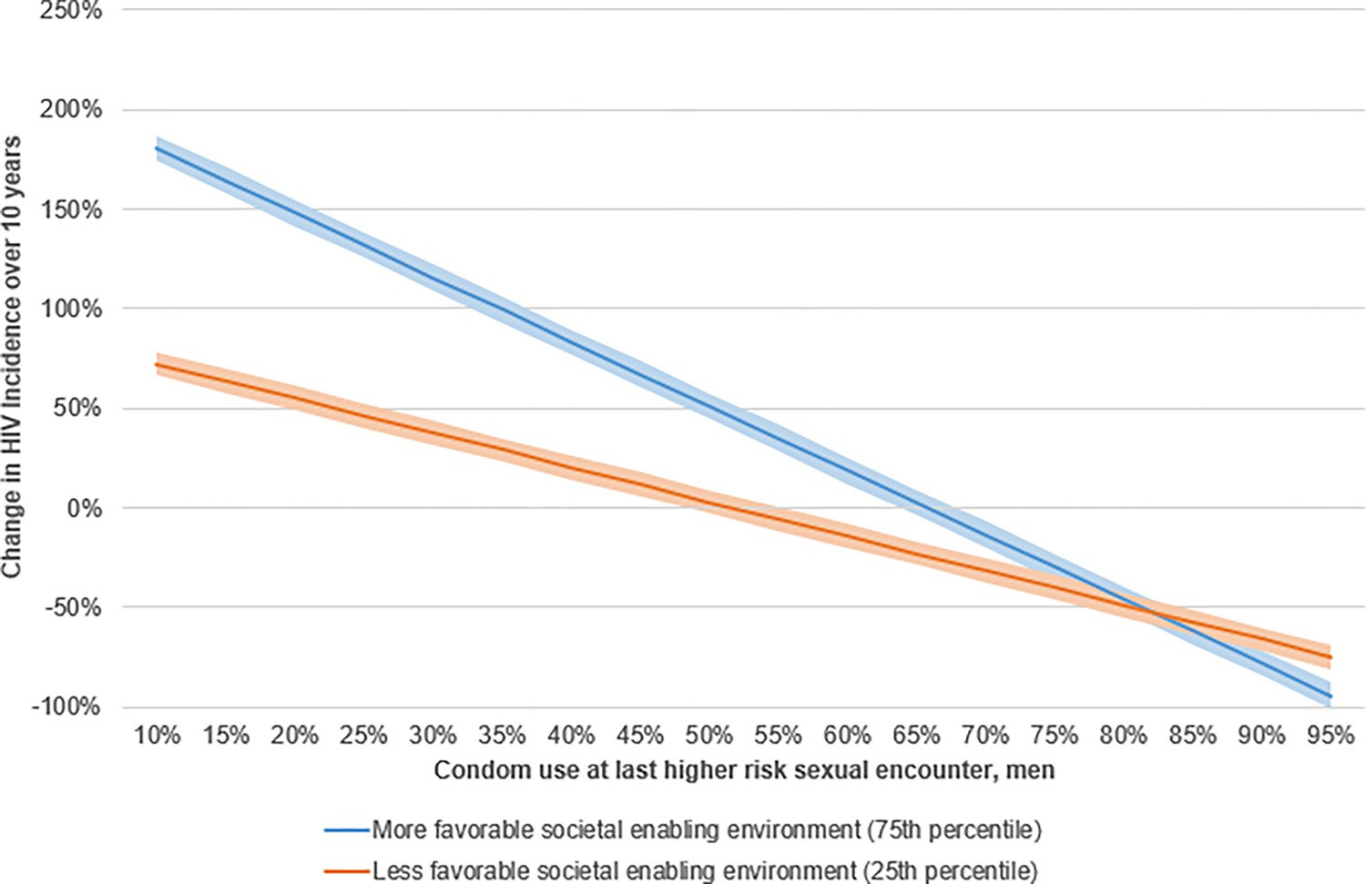
Moderating effect of unfavorable societal enabling environment on the relationship between HIV incidence decline and condom use (2010–2021).

Fig 6 illustrates the role played by the unfavorable societal enabling environment in the relationship between condom use and the change of HIV incidence over the past 10 years. The change in the rate of HIV infection in countries with low levels (as low as 10%) of condom use and a more favorable societal enabling environment was higher than in those with less favorable environments. Beyond a certain level of condom use (~82%), a more favorable S.E.E. leads to a bigger change in HIV Incidence. For the same absolute change in incidence, the percentage change would be more significant if the initial value was lower. For example, in Zambia HIV incidence per 1000 in 2011 and 2021 was respectively 8.184 and 5.212, while Romania in the same years had values of 0.049 and 0.046. This implies that Zambia’s HIV incidence change over 10 years in 2021 is -36.3%, while for Romania it is -6.1%. It is important to note that Zambia in 2021 had a 113 times higher HIV incidence than Romania. Furthermore, the scale of percentage change is not symmetrical, for example going from 6 to 3 is a -50% change, while going from 3 to 6 is a +100% change. The baseline levels of HIV incidence significantly differed between countries with higher and lower ranked societal enabling environments. Countries in the top 50% in terms of the societal enabling environment had an average incidence of 1.14 per 1000 population in the period between 2000 and 2009. On the other hand, countries in the bottom 50% had an average incidence of 2.10 per 1000 population for the same period.

**Fig 7:**
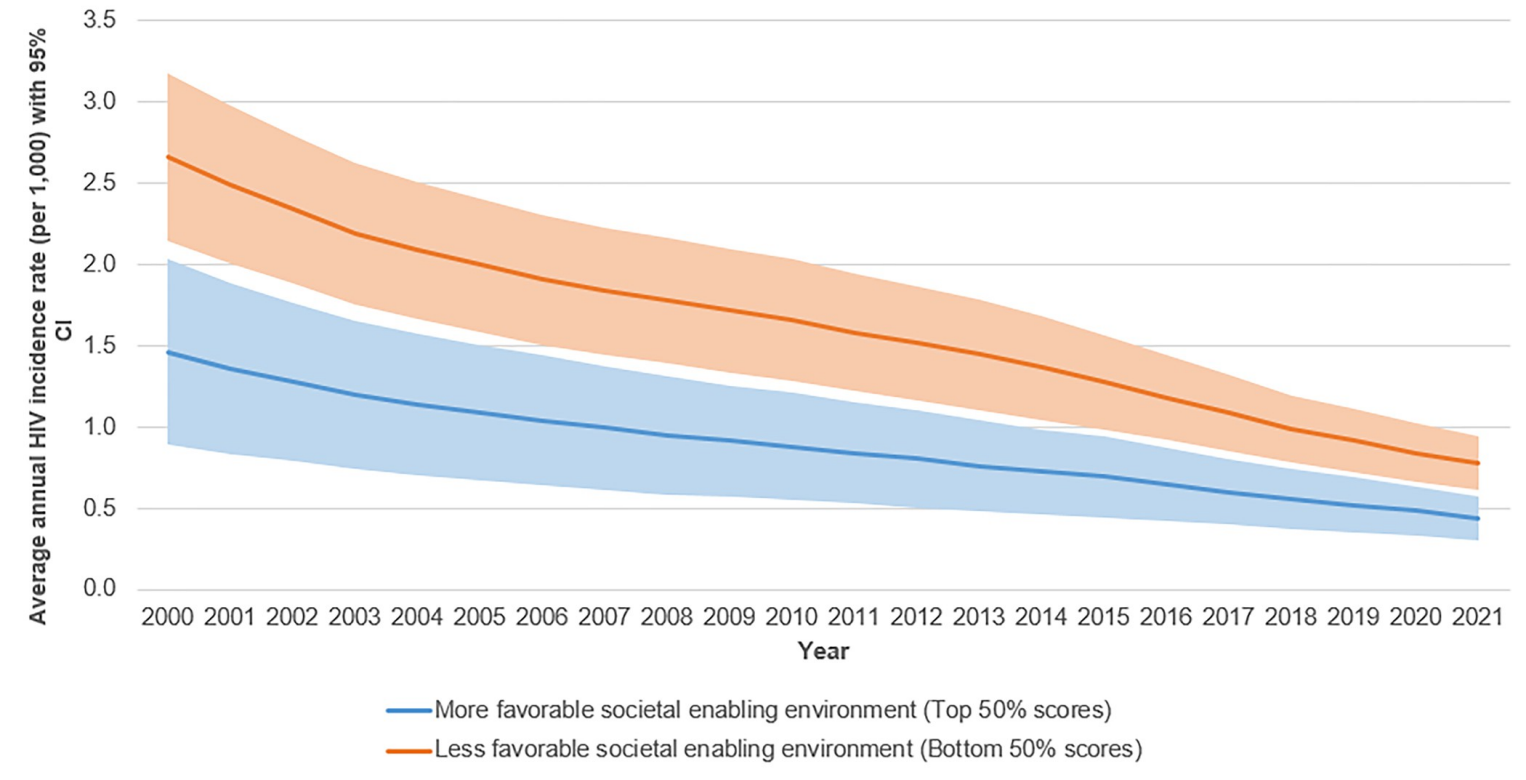
Average annual HIV Incidence rate (per 1,000) with 95% confidence intervals for countries in the top 50% and in the bottom 50% of scores for societal enabling environments, 2000–2021.

Fig 7 illustrates an overall comparison of HIV rates among countries. There were significant disparities among the top 50% and the bottom 50% of countries ranked by societal enabling environment scores. Countries with higher ranked societal enabling environments had much lower levels of HIV incidence per 1000 at the beginning of the period, approximately three times less than countries with lower ranked enabler scores. This disparity would explain the larger percentage changes for countries with higher ranked societal enabling environments, especially those with low levels of condom use. HIV rates would be likely to increase everywhere when condom use was low. Moreover, the absolute change would likely prove similar for both groups of countries.

### Direct and indirect effects of societal enablers from SEM

Table 1 below shows the direct and indirect effects of the unfavorable societal enabling environments on HIV outcomes. Ninety-five percent bias-corrected confidence intervals were calculated by performing bootstrap. It was found that both the direct and indirect standardized effects of unfavorable societal enabling environments showed a positive association with AIDS-related mortality among PLHIV (0.26 and 0.08, respectively). These results suggest that unfavorable societal enabling environments affect AIDS-related mortality both directly as well as indirectly through HIV programmes. The negative sign of direct standardized effect of an unfavorable societal enabling environment on HIV incidence change (-0.30) could be explained by the difference in the average HIV incidence levels between the top and bottom 50% of countries ranked by societal enabling environment (see Fig 7).

**Table 1:** Effects of unfavorable S.E.E. on HIV outcomes.

	Standardized Effects of Unfavorable Societal Enabling Environment	95% Confidence interval
**Direct effect**		
AIDS related mortality among PLHIV	0.26	(0.25, 0.27)
HIV Incidence: change over 10 years	-0.30	(-0.31, -0.29)
**Indirect effect**		
AIDS related mortality among PLHIV	0.08	(0.07, 0.09)
HIV Incidence: change over 10 years	0.19	(0.18, 0.20)
**Total effect**		
AIDS related mortality among PLHIV	0.34	(0.32, 0.36)
HIV Incidence: change over 10 years	-0.11	(-0.13, -0.09)

The indirect effect of an unfavorable societal enabling environment on HIV incidence change through HIV programmes has a positive sign (+0.19), indicating that as the environment worsens, the incidence change would increase more. An increase in HIV incidence change means that HIV incidence increases over time or decreases slower than it would if the societal environment was more favorable. For example, an unfavorable societal environment might have indirectly caused HIV Incidence to decrease only 25% instead of 50%, or even to increase 10% instead of decreasing 10% in the ten years span compared to a more favorable societal enabling environment.

The direct and indirect effects of each of the four societal enablers were also calculated separately. However, none of the enablers demonstrated independent statistical significance. This suggests that the moderation of the societal enablers on programme effectiveness only occurs when the entire societal enabling environment was addressed.

### Multi-group moderation analysis

Finally, the differential impacts of the composite societal enabler on relationship between HIV programmes and HIV outcomes were analyzed by separating the countries into two groups based on composite score values that were above or below the median score. The differences in the general effects of HIV programmes on HIV outcomes between the two groups were confirmed through the Chi-squared test. A comparison of the standardized coefficients of the ART coverage to AIDS-related mortality among PLHIV revealed a significant difference between the two country groups. This result suggested that the association of ART programmes with AIDS-related mortality was 50% stronger in countries with higher ranked societal environments than countries with lower ranked societal enabling environments (-0.61 and -0.39, respectively). However, there was no significant difference (*p*-value = 0.877) between the standardized coefficients of condom use and change in the incidence of HIV. The results indicated that the countries’ different ranks according to unfavorable societal enabling environments did not alter the impact of condom use on changes in the incidence of HIV.

## Discussion

This study shows the results of the SEM statistical analysis which formally tested the hypothesis that HIV related societal enablers as an enabling environment moderate the impact of HIV/AIDS service delivery, such as ART and condom use, on HIV outcomes (i.e., AIDS related mortality and HIV Incidence change). The analysis explored the direct and indirect role of HIV societal enablers as unobserved or latent constructs which compose an enabling environment and its moderation effects on HIV outcomes and overall programme-effectiveness. The results of this statistical analysis suggest that countries with better enabling societal environments correlate with lower levels of AIDS-related mortality when ART coverage level is held constant. However, due to high discrepancies in starting levels of HIV incidence between groups of lower and higher societal enabling environment, mixed results are achieved on the impact of societal enablers on changes in HIV incidence. The global response to HIV has three zeros in its long term vision: 1) zero HIV infections, 2) zero AIDS-related deaths, and 3) zero discrimination. The vision of zero discrimination has also been frequently seen as part of a rights-based approach [16]. Global bodies that advise on the global response to HIV, such as the UNAIDS Human Rights Reference Group, have promoted a rights-based approach for the response to HIV and supported the adoption and promotion of societal enablers as part of an effective response [17]. The UNAIDS updated Framework [3] on the role of societal enablers includes four components, which correspond to our definition of societal enablers. The study presented in this paper is part of a series of papers in the Public Library of Science (PLoS) journals and supported the 2025 HIV target setting process [2]. It attempts to demonstrate evidence that poor societal enablers increase HIV mortality and incidence and reduce overall programme effectiveness.

A number of studies have demonstrated the isolated effects of individual HIV related societal enablers on HIV outcomes and programmes [18–34]. Previous studies tested the connections between interventions, programmes, or baseline situations with HIV-related outputs and outcomes in specific country settings. One study has quantitatively shown the impact of societal enablers on HIV programme effectiveness [6]. However, few attempts have been made to assess the moderation of HIV programmes by societal enablers, either individually or combined as an overall societal enabling environment. The extensive literature review particularly identified significant gaps in systematic, statistically robust and evidence-based studies related to the joint effects of societal enablers on HIV outcomes and programme effectiveness.

Initial exploration as part of this study failed to produce statistically significant results to show that any of the societal enablers on their own or their components may not have direct association to HIV outcomes. Meaningful correlations were found in the literature review between elements of the societal enablers and intermediate level outputs of the HIV programmes. For example, there was a correlation between prevalent HIV-related stigma and discrimination and lack of testing or retention on ART [19–22].

The HIV-related societal enablers are unobserved constructs that have not been quantitatively defined. Structural equation modeling that combines factor analysis to create composite measures and address missing data, multiple regression, and path analyses have proved to be a compelling approach. Longitudinal or panel data were used to strengthen the non-experimental approach to causal analysis [35]. This approach permits the quantification of direct and indirect effects between observed variables and unobserved constructs and to define the effects in one or more dependent variables simultaneously.

The methodology used is appropriate to the slow speed of change in the societal enabling environment and also reflects the time lag between when a societal enabling environment is measured and when it may influence HIV outcomes. This study’s findings indicate that the lack of or slow progress in the improvement of the societal enabling environment is associated with higher AIDS-related mortality as well as slower and less change in the incidence of HIV over a 10-year period, an effect which is more marked with higher levels of condom use. The analysis also indicates that only when there is improvement in all the four enabling areas analyzed, there would be enhanced progress in HIV outcomes associated with HIV programme scale-up as there was no statistically significant effect observed when the societal enablers were tested separately.

The most significant challenge encountered in this study was the paucity of data per country in the different domains that describe each of the societal enablers. Most of the available data on the effect of any societal enabler was derived from pilot or small-scale studies conducted in selected countries, regions, or subnational areas or facilities.

Indicators to track the enablers per earlier framework of 2011 [1] were not always collected or reported by all countries every year [36] nor were all the indicators included in the UNAIDS Global indicator database. The framework was recently updated and it was included in the current Global AIDS strategy: End inequalities. End AIDS 2021–2026 [18] that was endorsed in the 2021 Political Declaration of the High-Level Meeting on ending AIDS [37]. The adoption of the strategy and the political declaration can contribute to strengthening the monitoring system specifically for societal enablers that in turn will allow improved data availability and better modeling of the relationships between unfavorable societal enabling environment and HIV outcomes.

An additional factor concerns the nature of the epidemiological variables. The incidence and mortality are projected data from the GOALS model rather than actual data, as surveillance system reports from countries yield incomparable data or are non-existing (e.g. HIV incidence). Similarly, and more specifically, the appropriate modeling of the relationship between condom use and HIV incidence constituted a challenge due to data availability and decision to mimic GOALS failing to include other programme data. The reducing effect of HIV-viral suppression (U = U) on HIV transmission could not be included in the model due to lack of data. In future modeling exercises, data on individual behavior among key populations on consistency of condom use could be added. It is desirable that data be systematically collected on other methods to reduce incidence, including behavioral risk reduction, partner reduction, VMMC, PrEP, PEP, U = U and viral suppression due to effective use of ART, so that it could be incorporated in future models on this same topic.

A subsequent set of analyses should include more variables on preventative programmes, including the percentage of total people living with HIV (or percentage of those on ART) who are virally suppressed. These analyses could better inform on the relationship between HIV programmes and HIV outcomes mediated by societal enablers as more variables on societal enablers are being implemented as a result from the 2021 United Nations High Level Meeting on AIDS and the 2021–2026 global HIV strategy.

External and content validity could be questioned given these constraints. However, the strategy to impute missing values with the methodology chosen helped to address some of these concerns. Nevertheless, statistical significance was a challenge for some aspects given that more dimensions of each of the societal enablers could not be included in the analysis. This concern proved particularly relevant when it became clear that results only showed statistical significance when all four societal enablers were modeled as a composite against each of the HIV outcomes, but not when the associations were tested against individual societal enablers separately.

Despite these challenges, the moderation effect of the unfavorable societal enabling environment between two variables—antiretroviral treatment and AIDS-related mortality—were found to be statistically significant. However, the correlation between condom use and 10-year HIV-incidence change did not show such a strong relationship. Explanations for the weaker relationships could include the complexity of human behavior in condom use, including frequency of condom use and type of partners, a negative association of condom use more frequently among PLHIV than those who know they do not live with HIV or ignore their status; or other confounding variables not accounted for in the 10-year period.

The direct association between the use of ART and lower AIDS-related mortality was observed in the 138 low- and middle-income countries modeled. An assessment of the hypothesized moderating role of the unfavorable societal enabling environments revealed that there was an independent and statistically significant moderation. The moderating role was evident even after controlling for potentially confounding variables, such as the human development index of countries (United Nation Development Programme, Human Development Reports), including levels of education, income, and CD4 cell counts upon ART initiation.

The results in this study demonstrated that the same increase in ART coverage results in greater decrease of AIDS-related mortality in countries with a more favorable societal enabling environment. Both the direct and indirect standardized effects of unfavorable societal enabling environments to AIDS-related mortality among PLHIV are statistically significant and positive (0.26 and 0.08, respectively). However, due to high discrepancies in starting levels of HIV incidence between groups of lower and higher societal enabling environment, mixed results are achieved on the impact of societal enablers on changes in HIV incidence.

The differences were observed at the 2019 average level of observed ART coverage of 59% as well as at the desirable 2025 target level of 90%. The associated AIDS-related mortality level would be much higher in countries with lower ranked societal enabling environments. If countries were to reach 90% ART coverage, the AIDS-related mortality among PLHIV would be three times higher for the lower ranked countries than the countries with higher ranked environments (1.47% and 0.49%, respectively).

The results showing significant associations between societal enabler variables on HIV outcomes on the one hand, and programme variables interacting with the societal enabler on the other, cannot be necessarily interpreted as causal relations, although they could very well reflect underlying causal mechanisms.

The relationship between the unfavorable societal enabling environment and HIV outcomes are of the utmost importance for the response to HIV in the next decade, both globally and in individual countries. The level of HIV programme effectiveness and levels of efficiency have been seriously questioned. The world failed to achieve the 2020 targets, primarily due to low levels of resource mobilization and a lack of rapid scale-up of services. This was evidenced in the failure to reach the global target goal of 500,000 or fewer new infections among adults annually by 2020. UNAIDS estimated that 1.7 million people were newly infected in 2019. Advocates have pointed out that alleged defunding of prevention activities in favor of testing and treatment may have been the reason. Others have argued that the rampant high levels of stigma and discrimination, punitive laws that criminalize populations at the highest risk to acquire HIV, and gender inequality—in particular gender-based violence—impeded efforts to reach this target. Additionally, the continued unsuccessful attempts to overcome these obstacles could negatively impact efforts to reach the 2030 goals and will make the process much more difficult and costly. The human cost would prove particularly devastating.

UNAIDS has argued that a failure to improve the societal enabling environment would undermine efforts to achieve the 2025 HIV service targets, and could result in 440,000 additional AIDS-related deaths, missing the opportunity to avert 2.6 million new HIV infections between 2020 and 2030 [38]. This failure would eventually undermine efforts to achieve the 2030 Sustainable Development Goals which include ending AIDS as a global public health threat. The modeled impact of societal enablers demonstrated the challenges to programmatic coverage targets; stigma, discrimination, criminalization of HIV risky behaviors, and gender-based violence act as impediments to access to HIV testing as well as adherence to antiretroviral treatment or preventative measures.

Societal enablers are complex unobserved constructs that vary significantly depending on cultural, religious, political, educational, and traditional values and have diverse manifestations. The societal enablers can be modeled using observed (i.e., reported or estimated) available data as proxy variables of their different components. The degree to which different countries embrace the changes needed to improve the societal enabling environments vary as there is opposition to programs focused on societal enablers because of reluctance to enforce human rights in general or for some populations. The findings from this study indicate that country choices resulting in specific societal enabling environments matter in the health of populations, at least with regards to the effectiveness of programmes in the prevention of HIV infections and the effort to save lives.

UNAIDS has compiled a list of 41 comprehensive programme options as guidelines for countries to improve the societal enabling environment. The intensity and combination of the specific programmes implemented are based on the baseline situation [39]. The major donor organizations in the HIV field, the United States President’s Emergency Plan for AIDS Relief (PEPFAR) and the Global Fund for AIDS, Tuberculosis, and Malaria have invested a significant amount of resources for the implementation and evaluation of these programmes. UNAIDS estimated that approximately US$1 billion was invested by the end of 2019 in programmes related to human rights, reduction of HIV stigma and discrimination for key populations, and access to justice. However, many of the programmes implemented today are not necessarily those that have shown proven impact. There may be a need to perform an in-depth review of the programmes currently implemented and reallocate the existing resources to those with proven efficacy. Those programmes should be funded appropriately with US $3 billion by 2025 in order to improve the societal enabling environment and remove existing impediments to optimal HIV programme effectiveness [36].

The recently adopted UNAIDS Global AIDS strategy: End inequalities. End AIDS 2021–2026 [18], the report of the United Nations Secretary General to the 75th United Nations General Assembly on the Implementation of the Declaration of Commitment on HIV/AIDS [37], and other recent political declarations on HIV/AIDS recognize the failure to achieve the 2020 targets. Moreover, they indicate that one reason for this failure has been a lack of progress in the societal aspects of responses to HIV, recognizing the need to address societal enablers, including co-action with the broader dimensions of these enablers that appear in the Sustainable Development Goals. These documents also called for a framework that would help to monitor progress in the implementation of societal enablers by all countries on a yearly basis. The research agenda would include the generation of robust data to represent the content of each of the societal enablers using programme data. A broader database that includes more countries would permit innovative analyses that could retest and reconfirm the hypothesis examined in this study and improve precision in the estimates of the impacts of societal enablers on HIV outcomes. Countries should be strongly encouraged to invest in improving data collection and data quality, so that future research can corroborate the present findings.

Finally, the evidence presented here could support the decision-making process with regard to the planning, advocacy and actions taken to improve programme effectiveness and efficiency in the use of the resources available. The removal of stigma and discrimination, increased access to justice, law reform, and progress toward gender equality would improve the response to HIV in addition to their own contribution to a better world. Moreover, these efforts would help to reach a sustainable response sooner, which would reduce the health, social, and economic costs. Initiatives that integrate societal enablers should represent key priorities for global, regional, national and subnational AIDS programmes.

## Supporting information

S1 Table Description of the indicators of the societal enablers and relevant HIV programmes and outcomes used in the model, classification, applicable population, data source, missing data and imputed percentage, and theoretical basis. (XLSX)

S2 Table Description of the indicators of the societal enablers considered, but not used in the model, classification, applicable population, data source, missing data and theoretical basis. (XLSX)

S1 List Countries/Territories included (138) in the final model. (DOCX)

S1 Data Dataset used for CFA model. (XLSX)

S2 Data Dataset used for SEM model. (XLSX)

## List of recurring abbreviations

AIDS: Acquired Immune Deficiency Syndrome
ART: Antiretroviral Treatment
AVE: Average Variance Extracted
CFA: Confirmatory Factor Analysis
CFI: Comparative Fit Index
CMIN/DF: Chi-Square/Degrees of Freedom
CR: Composite Reliability
FIML: Full Information Maximum Likelihood
GAM: Global AIDS Monitoring
PEPFAR: President’s Emergency Plan for AIDS Relief
PLHIV: People Living with HIV/AIDS
PMTCT: Prevention of Mother to Child Transmission
POPART: Population Effects of Antiretroviral Therapy to Reduce HIV
PrEP: Pre-Exposure Prophylaxis
RMSEA: Root Mean Square Error of Approximation
SE: Societal Enabler
SEE: Societal Enabling Environment
SDG: Sustainable Development Goals
VMMC: Voluntary medical male circumcision
