# Supplementary material for: Exploring relationships between HIV programme outcomes and the societal enabling environment: A structural equation modeling statistical analysis in 138 low- and middle-income countries
Source: PLOS Glob Public Health. 2023 May 9;3(5):e0001864. doi: 10.1371/journal.pgph.0001864 (PMC10168546; doi:10.1371/journal.pgph.0001864)
Supplement: S1 List — (DOCX) [file pgph.0001864.s003.docx]

S1. List Countries/Territories included (138) in the final model:

Afghanistan, Albania, Algeria, American Samoa, Angola, Argentina, Armenia, Azerbaijan, Bangladesh, Belarus, Belize, Benin, Bhutan, Bolivia Plurinational State of, Bosnia and Herzegovina, Botswana, Brazil, Bulgaria, Burkina Faso, Burundi, Cambodia, Cameroon, Cape Verde, Central African Republic, Chad, China, Colombia, Comoros, Congo, Congo the Democratic Republic of the, Costa Rica, Cuba, Côte d’Ivoire, Djibouti, Dominica, Dominican Republic, Ecuador, Egypt, El Salvador, Equatorial Guinea, Eritrea, Ethiopia, Fiji, Gabon, Gambia, Georgia, Ghana, Grenada, Guatemala, Guinea, Guinea-Bissau, Guyana, Haiti, Honduras, India, Indonesia, Iran Islamic Republic of, Iraq, Jamaica, Jordan, Kazakhstan, Kenya, Kiribati, Korea Democratic People’s Republic of, Kyrgyzstan, Lao People’s Democratic Republic, Lebanon, Lesotho, Liberia, Libya, North Macedonia, Madagascar, Malawi, Malaysia, Maldives, Mali, Marshall Islands, Mauritania, Mauritius, Mexico, Micronesia Federated States of, Moldova Republic of, Mongolia, Montenegro, Morocco, Mozambique, Myanmar, Namibia, Nepal, Nicaragua, Niger, Nigeria, Pakistan, Palau, Palestine State of, Panama, Papua New Guinea, Paraguay, Peru, Philippines, Romania, Russian Federation, Rwanda, Saint Lucia, Saint Vincent and the Grenadines, Samoa, Sao Tome and Principe, Senegal, Serbia, Sierra Leone, Solomon Islands, Somalia, South Africa, South Sudan, Sri Lanka, Sudan, Suriname, Swaziland, Syrian Arab Republic, Tajikistan, Tanzania United Republic of, Thailand, Timor-Leste, Togo, Tonga, Tunisia, Turkey, Turkmenistan, Tuvalu, Uganda, Ukraine, Uzbekistan, Vanuatu, Venezuela Bolivarian Republic of, Viet Nam, Yemen, Zambia, Zimbabwe
